# Cellular Interaction of Human Skin Cells towards Natural Bioink via 3D-Bioprinting Technologies for Chronic Wound: A Comprehensive Review

**DOI:** 10.3390/ijms23010476

**Published:** 2022-01-01

**Authors:** Syafira Masri, Mazlan Zawani, Izzat Zulkiflee, Atiqah Salleh, Nur Izzah Md Fadilah, Manira Maarof, Adzim Poh Yuen Wen, Fatih Duman, Yasuhiko Tabata, Izhar Abd Aziz, Ruszymah Bt Hj Idrus, Mh Busra Fauzi

**Affiliations:** 1 Centre for Tissue Engineering and Regenerative Medicine, Faculty of Medicine, Universiti Kebangsaan Malaysia, Cheras, Kuala Lumpur 56000, Malaysia; eiramasri@gmail.com (S.M.); nurzawaniofficial@gmail.com (M.Z.); mizzatzulkiflee@gmail.com (I.Z.); atqhsalleh@gmail.com (A.S.); nurizzahfadilah@gmail.com (N.I.M.F.); manira@ppukm.ukm.edu.my (M.M.); yasuhiko@infront.kyoto-u.ac.jp (Y.T.); ruszyidrus@gmail.com (R.B.H.I.); 2 Department of Surgery, Hospital Canselor Tuanku Muhriz, Universiti Kebangsaan Malaysia, Cheras, Kuala Lumpur 56000, Malaysia; adzimpoh@gmail.com; 3 Department of Biology, Faculty of Science, University of Erciyes, 38039 Kayseri, Turkey; fduman@erciyes.edu.tr; 4 Department of Biomaterials, Institute of Frontier Medical Science, Kyoto University, 53 Kawara-cho Shogoin, Sakyo-ku, Kyoto 606-8507, Japan; 5 3D Gens Sdn Bhd, 18, Jalan Kerawang U8/108, Bukit Jelutong, Shah Alam 40150, Malaysia; izhar@3dgens.com

**Keywords:** 3D-bioprinting, cellular activity, precision medicine, bioinks, wound healing, biomaterials

## Abstract

Skin substitutes can provide a temporary or permanent treatment option for chronic wounds. The selection of skin substitutes depends on several factors, including the type of wound and its severity. Full-thickness skin grafts (SGs) require a well-vascularised bed and sometimes will lead to contraction and scarring formation. Besides, donor sites for full-thickness skin grafts are very limited if the wound area is big, and it has been proven to have the lowest survival rate compared to thick- and thin-split thickness. Tissue engineering technology has introduced new advanced strategies since the last decades to fabricate the composite scaffold via the 3D-bioprinting approach as a tissue replacement strategy. Considering the current global donor shortage for autologous split-thickness skin graft (ASSG), skin 3D-bioprinting has emerged as a potential alternative to replace the ASSG treatment. The three-dimensional (3D)-bioprinting technique yields scaffold fabrication with the combination of biomaterials and cells to form bioinks. Thus, the essential key factor for success in 3D-bioprinting is selecting and developing suitable bioinks to maintain the mechanisms of cellular activity. This crucial stage is vital to mimic the native extracellular matrix (ECM) for the sustainability of cell viability before tissue regeneration. This comprehensive review outlined the application of the 3D-bioprinting technique to develop skin tissue regeneration. The cell viability of human skin cells, dermal fibroblasts (DFs), and keratinocytes (KCs) during in vitro testing has been further discussed prior to in vivo application. It is essential to ensure the printed tissue/organ constantly allows cellular activities, including cell proliferation rate and migration capacity. Therefore, 3D-bioprinting plays a vital role in developing a complex skin tissue structure for tissue replacement approach in future precision medicine.

## 1. Introduction

The skin substitution approach has been extensively accepted for clinical use to enhance wound closure and promote normal skin function [1]. Dry wound dressing, including gauze and bandages, are widely used in the early stage of wound healing [2]. The goal of wound dressings is to promote wound closure, enhance new tissue formation, and reduce scar formation. Clinically, the autologous split-thickness skin graft (ASSG) remains a gold standard for extensive wound treatments. It involves taking a specific thickness of healthy skin from other patients and reapplying the ASSG onto the injury site [1]. However, patients with severe burns may not receive adequate skin grafts and are at a greater risk of acquiring infections, including hepatitis B or C [3]. Besides, another traditional approach for chronic wound therapy is via fish skin acellular treatment [4]. This method is considered as one of the significant treatments due to its histological properties that promote cellular regulation and is rich with omega-3 fatty acids to supply to the local tissue [5,6].

### 1.1. Wound Healing

**Figure 1:**
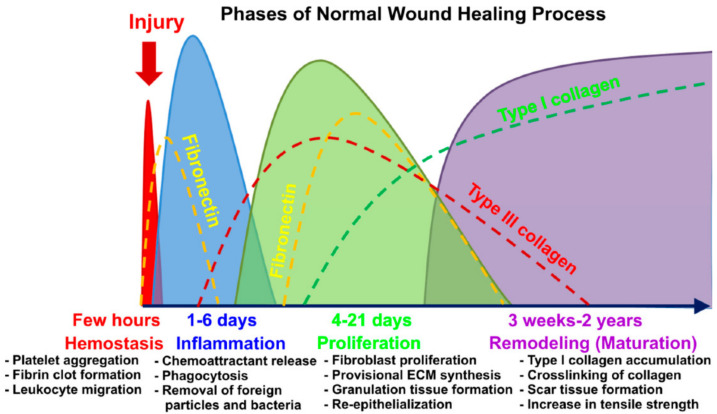
Graphical abstract for wound healing phases [12]. Used under the Creative Commons License (http://creativecommons.org/licenses/by/4.0/) accessed on 13 November 2021.

Wound healing is a dynamic and complex process that initiates the immune response for tissue repair [5]. Several types of wounds, including vascular ulcers, pressure ulcers, and diabetic ulcers, are primarily categorized as chronic wounds [6]. The abnormal pathological conditions of chronic wounds lead to a poor healing rate or excessive scar formation after recovery. Generally, the chronic wound is the most critical challenge related to skin problems. The wound healing phases start immediately after wound formation, followed by the inflammatory phase begins after the hemostasis phase is completed [7]. The hemostasis phase involves the activation of the enzyme precursors, which results in platelet aggregation at the wound site. Thus, the production of a fibrin clot (fibronectin and factor XIII) will be activated to prevent excessive blood loss [8,9]. Besides, the secretion of extracellular proteins, including plasma fibrinogen and fibronectin, promotes wound closure by accelerating cell migration, proliferation, and function [10]. Overlapping the hemostasis process, the inflammation phase helps to recruit the inflammatory cells to the wound area. In this cascade, the inflammatory cells will eliminate pathogens from the wound site and prevent severe complications. Within two to ten days of post-injury, the proliferation phase will take place, where new tissue formation begins with cell proliferation and migration of keratinocytes towards the lesion [11]. Finally, the tissue remodeling begins after several weeks of the injurious event and may last over more than a year [11]. During this phase, all of the essential cellular responses that were stimulated during injury are downregulated and eventually terminated [8]. Figure 1 shows the graphical abstract for wound healing phases, as discussed in the review paper of A.Przekora (2020) [12].

**Figure 2:**
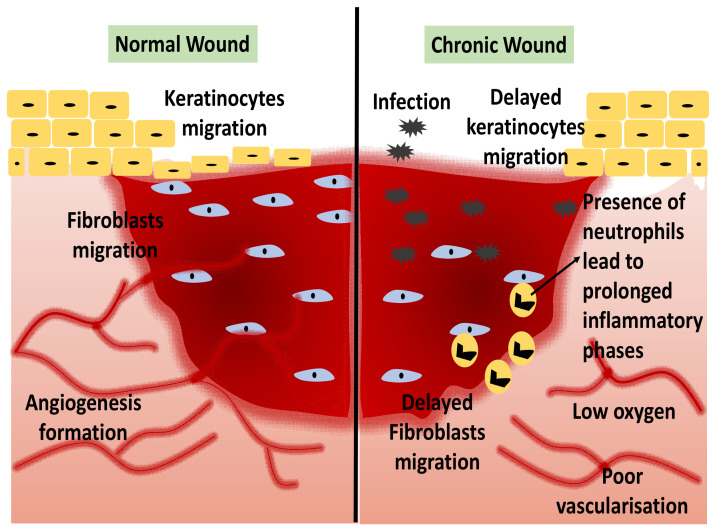
A comparison between normal and chronic wounds.

Chronic wounds are more likely to occur by sustained stimulation, such as hyperglycemia, chronic inflammatory responses, or persistent tissue injury [13]. Non-healing wounds fail to complete the entire wound healing stages and usually have prolonged inflammatory phases. Interruption of the normal healing phase may result in additional phases of a chronic condition, which may indirectly increase the patient’s vulnerability to infection and, ultimately, damage the patient’s quality of life [14]. Problematic wound healing can occur due to a wide range of health conditions and pathologic developments, including chronic inflammation, persistent infections, “open wounds”, and cancerous wound transformation [15]. Diabetes mellitus (DM) has a serious complication that might result in diabetic foot ulcers (DFU). DFU has been related to poor wound healing progress due to cytokines and poor cellular responses, infections, poor vascularisation, and diebetic neuropathies [16]. The primary goal of wound healing is to prevent the wound from being infected by the pathogens from the external environment [17]. Thus, the neutrophil influx is an early inflammatory response required for the clearing of pathogens and cellular debris during cutaneous wounds [18]. Hence, faster wound repair is vital for wound healing treatment. Figure 2 shows the comparison of normal and chronic wound conditions.

Tissue engineering has proposed a combination of cells, biomolecules, and biomaterials approach to replace the conventional skin graft. The complex structure of skin tissue requires a combination of several types of elements to form a biocompatible scaffold that mimics the native tissue. Thus, three-dimensional (3D) bioprinting is an innovative fabrication technique that combines selected cells with “inks” composed of biomaterials, crosslinkers, and growth factors to fabricate tissue-like structures for various applications. On the other hand, the use of 3D-bioprinted technology decreases the number of operations necessary for skin replacement. The 3D-shaped bioscaffolds open up new alternatives, such as broadening the range of structures accessible to treat injured skin tissues [19]. It allows for the precise placement of skin cells to replace damaged skin [20]. The bioscaffold has the potential to generate better properties for skin constructs with good elasticity, extensibility, and a high yield of skin reconstruction [1]. The network of blood vessels may be printed as well to ensure the long-term survivability of the skin tissue.

### 1.2. Current Trend of 3D-Bioprinting for Chronic Wound

**Figure 3:**
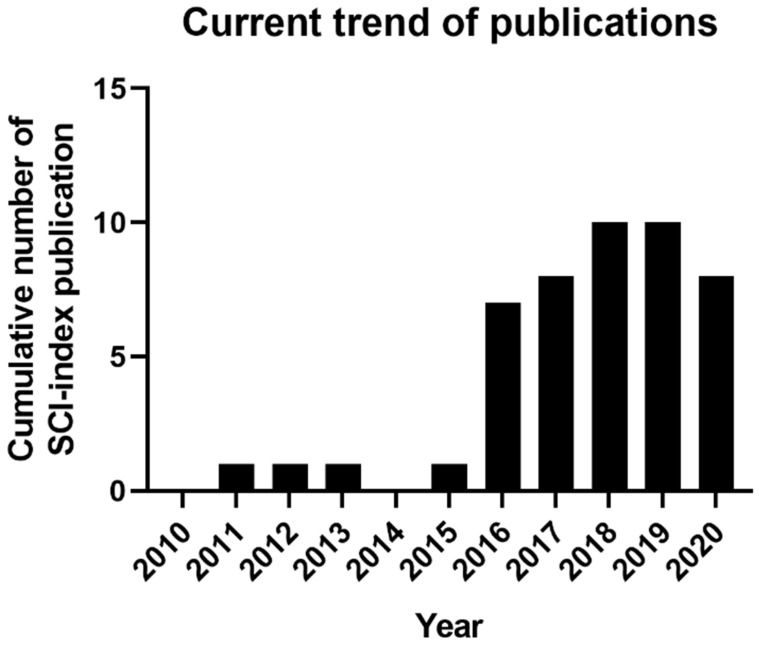
The current trend of SCI-indexed publications on Web of Science for chronic wound healing treatment by using a 3D-bioprinting approach.

Although skin has a highly complex structure, bioprinting techniques are the most reliable and convenient transfer of cells with accurate printing outputs and mimic native skin tissue [21]. In skin tissue engineering, 3D-bioprinting is continuously changing as researchers innovate and propel the field ahead. The recent trend in using the 3D-bioprinting approach for chronic wound healing treatment is still under study with several limitations. Figure 3 shows the current trend of the publications for chronic wound healing treatment by using a 3D-bioprinting approach from the year 2010 until 2020. A comprehensive search strategy was followed to collect the digital publication records on Web of Science. The search was limited to articles published from the year 2010 until 2020. The search query consists of seven terms including “3D-bioprinting”, “bioinks”, “three-dimensional”, “tissue engineering”, “skin cells”, “skin regeneration”, and “wound healing”. The publication summary (Figure 3) indicates that the research for chronic wound healing treatment by using 3D-bioprinting was highest in the years 2018 and 2019 compared to the previous eight years. The researchers used different types of biomaterials as bioinks. However, most of the biomaterial entails certain limitations, and the bioinks used successfully met the skin cells’ ideal conditions, including dermal fibroblasts (DFs) and keratinocytes (KCs).

Upon reaching the year 2020, 3D-bioprinting worldwide are still lacking effective wound dressings targeted for chronic wound therapy. This phenomenon is projected to rise for wound care products in the wound dressing market [22]. In general, the concept of skin 3D-bioprinting was established by researchers worldwide by extracting a sufficient number of cells from skin tissue culture after biopsy. The cells are then appropriately mixed with the specified biomaterials (bioinks) prior to being printed out using a 3D bioprinter to develop customized skin. Skin bioprinting can be performed using in vitro and in situ bioprinting techniques. However, both approaches have similar printing mechanisms except for the printing site and tissue maturation [23]. 

## 2. Human Skin Structure

**Figure 4:**
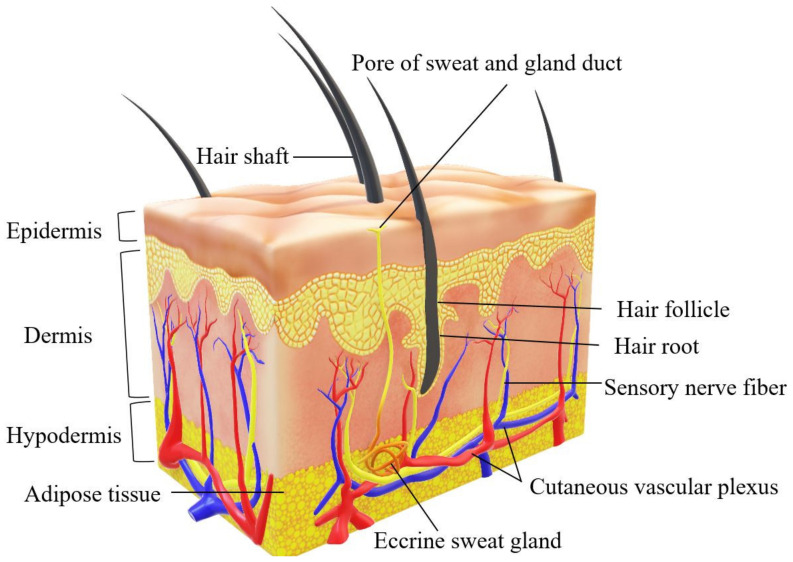
Complex human skin structure (epidermis, dermis, and hypodermis).

Skin is the largest organ of the human body with three different complex layers (epidermis, dermis, and hypodermis) and several other components, including the extracellular matrix (ECM), blood capillaries (veins and arteries), nerves, and hair follicles [12]. It is essential for maintaining skin integrity and stability for appropriate function in retaining body homeostasis [24]. Figure 4 shows the illustration of the complexity of human skin structure. 

The epidermis layer is abundant with keratinocytes to protect the skin from external infections, whereas the dermis layer acts as the skin’s appendages [25]. The dermis is made up of fewer cellular constituents, primarily fibroblasts [26]. The dermis layer lies within a complex connective tissue structure occupied with nerves, hair follicles, glands, and blood vessels for nutrient transportation [21]. Dermal fibroblasts (DFs) are the most abundant cells that occupy the dermis layer of the skin [27]. The dermis is composed of two connective tissues that interact to form an interconnected network of collagenous and elastin fibers produced by DFs [28]. The well-vascularisation inside the dermal layer will supply nutrients to the DFs. In the skin, DFs are responsible for the secretion of growth factors and extracellular matrix (ECM) for tissue regeneration [29]. The subcutaneous tissue, or hypodermis, is a fibrofatty layer that is loosely connected to the dermis layer of the skin [30]. The hypodermis is mainly composed of adipose tissue, which serves as an energy storage and insulation system for the body as well as a cushion for the skin. A muscle layer can be found adjacent to this layer, which overlies either bony prominences or interior tissues and organs. It is also the site of the formation of certain blood vessels that extend into the dermis [31].

### Skin Tissue Engineering

Limitations in skin grafting for wound healing have drawn researchers to set their sights on skin tissue engineering as an alternative chronic wound treatment. The main idea of tissue engineering is to incorporate scaffolds, cells, and bioactive molecules to promote the skin in regenerating from injuries [32]. However, the usage of a scaffold in tissue engineering has become the main challenge because it may have a significant impact on cellular activities [33]. Previously, traditional fabrication techniques such as electrospinning, freeze-drying, and gas-foaming needed longer to construct a complex scaffold that mimics human skin tissue. Besides, they provide excellent efficiency in tissue regeneration and remodeling. It provides a more systematic and accurate structure of artificial skin construct that can enhance cellular interactions with the skin layers [3]. At the moment, 3D-bioprinting offers a great degree of flexibility and reproducibility by employing a computer-controlled 3D printer to create 3D objects via layer by layer printing process [1]. The first step in skin bioprinting is tissue imaging for reconstruction through computed tomography (CT) or magnetic resonance imaging (MRI). As a new branch of 3D-bioprinting, it offers different printing techniques, including extrusion-based bioprinting, inkjet-based bioprinting, and laser-based bioprinting [24]. Briefly, the first bioprinting attempt was by using the inkjet bioprinting approach. However, the initial problem encountered was the cell viability significantly reduced during printing due to the instantaneous drying out of the hydrogel [34]. 

In addition, another option for 3D-bioprinting is through the laser-based bioprinting approach. However, this bioprinting technique leads to cell damage through the use of UV light and a photoinitiator. Nowadays, researchers have investigated an alternative technique to enable the photopolymerization of bioinks using a suitable UV spectrum. Besides, extrusion-based bioprinting offered the most favorable bioprinting technique that can replace the usage of UV light. However, it requires high viscosity of bioinks for a decent printing result. The low viscosity of hydrogel will produce a poor structure of the bioscaffold that leads to poor shape fidelity. Generally, the extrusion-based 3D printing technique employs a pneumatic actuator or screw mechanism to push material through a cartridge and into a nozzle for deposition of bioinks [24]. These common extrusion techniques are compatible with a wide range of materials. Hydrogels are primarily non-Newtonian fluids; their viscosity varies with shear rate. The high viscosity of hydrogel will generate high shear stress during printing, resulting in increased cell apoptotic activity [34]. To date, extrusion-based bioprinting is currently feasible only by using extrusion-based bioprinting due to its mechanism that is extremely biocompatible with cell-laden hydrogel due to its excellent capability to print bioinks with high cell density [35]. Furthermore, the system is simple to implement and can be utilized by operators with little experience with the technology [1]. However, additional training for advanced bioprinting software might be needed to explore the complexity of bioprinting with various tissue types, i.e., bilayered skin structure with the integration of blood vessels and other components.

## 3. 3D-Bioprinting for Chronic Wound

Nowadays, in parallel with the advance of technology, direct printing of living cells and biomaterials have opened up new possibilities for 3D tissue engineering and regenerative medicine [36]. The final product for the 3D-bioscaffolds is in the form of a hydrogel. Hydrogels are widely perceived as one of the excellent wound dressings [37]. The selection of bioinks must meet certain criteria, including printing resolution, gelation, viscoelasticity, mechanical properties, and biocompatibility to maintain the viability of the cells upon bioprinting [38]. The interaction of cells with the components of the bioinks needs to be considered for developing a harmoniously organized tissue [39]. Previously, the generation of autologous single-layer keratinocytes, single layer fibroblasts, and bilayer skin in prior work (MyDerm^TM^) was successfully implanted in patients [40]. The success of this work has proven that tissue replacement can be accomplished by using the patient’s cell with a combination of autologous biomaterial. Besides, this approach also eliminated the risks of immune rejection upon post-implantation failure. Consequently, it is preferable to use a biomaterial that maintains a homogenous solution of encapsulated cells with minimal cell sedimentation [37].

### 3.1. In Vitro Skin 3D-Bioprinting

**Figure 5:**
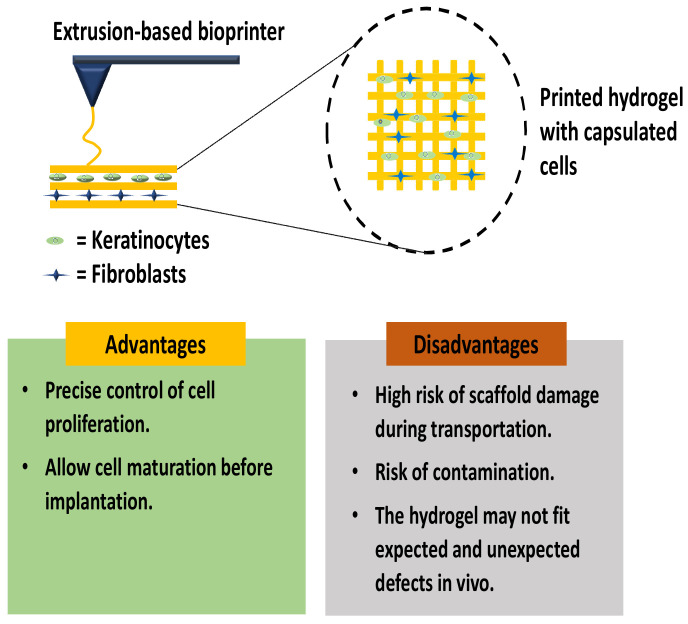
In vitro 3D-bioprinting using extrusion-based bioprinting.

In vitro skin bioprinting aims to improve the tissue maturation progress before transplantation to the wound site is performed [23]. As a result, this approach allows rapid wound healing progress and tissue regeneration. The usage of appropriate bioinks allows the composite scaffold to achieve adequate pore sizes, improve mechanical strength, and optimize the biodegradation rate for future clinical applications [41]. The bioinks optimization step is designed to provide a cell-friendly environment that promotes cell proliferation rate. However, the most challenging aspect of skin bioprinting is to combine various types of cells in the bioinks for skin tissue reconstruction. Dermal fibroblasts (DFs) and keratinocytes (KCs) are the major cells involved in skin model development [42]. Figure 5 shows the in vitro 3D-bioprinting of the skin layer by using DFs and KCs at different layers. 

The 3D microenvironment is required to facilitate cell development and maturation. The DFs and KCs easily isolated from any healthy skin biopsies samples using the standard operative procedure. Skin tissue promotes oxygen transportations and nutrients to all surrounding tissue; it is critical for developing a new tissue/organ with a vascularized structure. Fortunately, 3D-bioprinting opens up new possibilities for constructing adaptable skin models with vascularization and complex macrostructures [42]. Some researchers are susceptible to using in situ skin bioprinting against in vitro bioprinting due to several limitations during the handling and implantation procedure. An in vitro skin bioprinting study discovered that certain reconstructed 3D-skin models exhibited significant fragile micro and macro-structures. This may result in structural impairments such as swelling, contraction, or distortion upon transplantation. Furthermore, in vitro bioprinting is subject to a significant risk of contamination during transportation and manual implantation [43].

### 3.2. In Situ Skin 3D-Bioprinting

**Figure 6:**
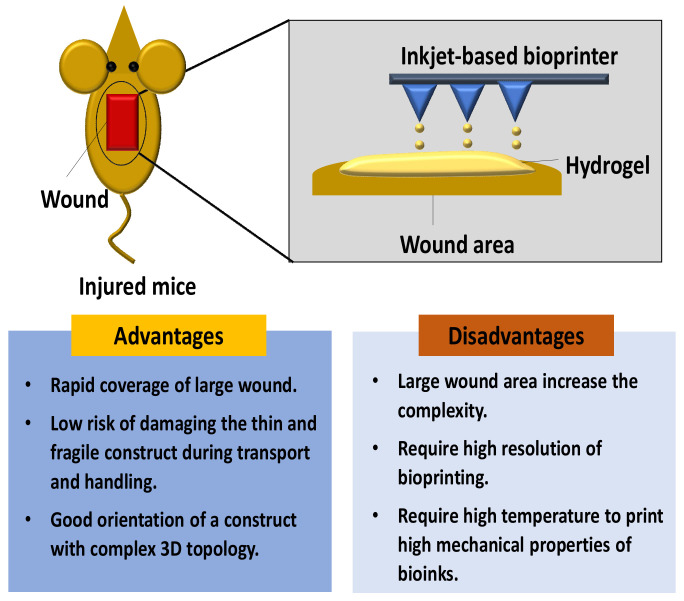
In situ bioprinting for the wound by using inkjet-based bioprinting technique.

To date, significant progress in tissue engineering has been proved by introducing in situ bioprinting technique. The basic principle for in situ bioprinting is performing a bioprinting method of pre-cultured cells directly onto the skin injury site and allowing for skin maturation at the wound area [23]. The in situ bioprinting approach provides a novel delivery bioinks approach for cell deposition at the injury site. Figure 6 shows the deposition of bioinks in a mouse wound by using the inkjet-based bioprinting technique. In situ bioprinting of the skin construct directly on the wound site is dependent on the patient’s body acting as a “bioreactor” for the functional maturation of the bioprinted tissue [44]. However, the wounds were scanned first to get accurate information on the wound topography, which was then used to direct the printing head to deposit the bioinks onto the injury site [45].

Overall, the laser wound scanner aids in the creation of a precise shape/map of the lost skin, and the bioinks will be printed out to this region [23]. The major advantage of the in situ bioprinting technique is that it facilitates the removal of artificial microenvironment formation, which is essential in newly formed tissue. In situ bioprinting approach provide rapid coverage towards the larger wound area [44].

## 4. Natural Biomaterials

**Figure 7:**
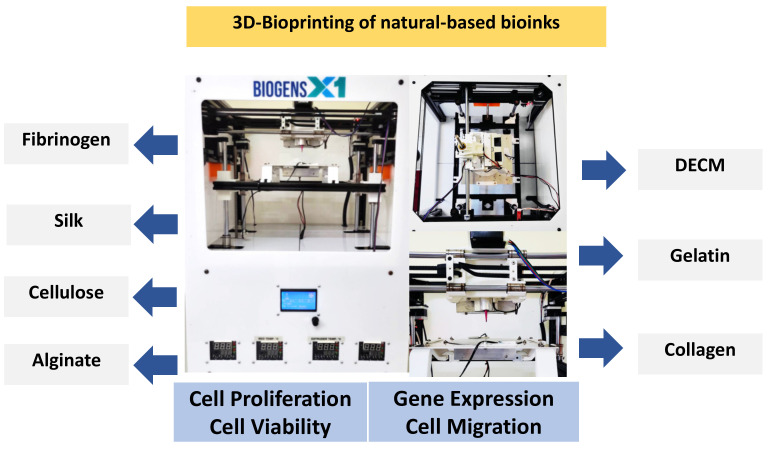
Cellular activities that the bioinks can influence.

A desirable property of bioinks should enhance the physicochemical properties, including the rheological, mechanical, chemical, and biological properties of the fabricated scaffold to mimic the native tissues. A hydrogel that resembles the composition of the ECM has received much attention. Natural-based bioinks have become the most favored bioinks for tissue engineering applications due to their non-immunogenic, biocompatibility, biodegradability, and hydrophilicity properties [46]. Table 1 summarizes the comparison of the biomaterial properties. On the other hand, synthetic-based bioinks provide better opportunities for tissue/organs construction [47]. The optimization of the bioinks should lead to an acceptable level of cellular activities, including cell migration, cell proliferation, cell viability, protein/gene expression, as in Figure 7.

### 4.1. Decellularised Extracellular Matrix

Several types of research currently utilize the decellularised extracellular matrix (dECM)-based bioinks for 3D skin bioprinting to encounter chronic wounds. The dECM bioinks are derived naturally from the human body, and it is known that our bodies are majority composed of extracellular matrix (ECM). The ECM is a non-cellular component that provides fundamental cellular processes, including cell spreading, growth, proliferation, migration, differentiation, and organoid formation [48]. Human ECM provides critical roles by providing structural support for tissue development with an ideal microenvironment [49]. The dECM preserves structural and functional features of the ECM, such as its nanostructure, biochemical complexity, and bioinductive capabilities [50]. Therefore, dECM bioinks could provide an excellent platform to promote cellular activities for skin regeneration. The main sources of dECM include tissue vascularisation factors like vascular endothelial growth factor (VEGF), platelet-derived growth factor (PDGF), and angiopoietins will improve the development of the 3D-bioprinting tissue model [40,44].

Decellularization is the process of removing all cellular components from a tissue scaffold of interest. There are several sources of mammalian dECM that are suitable for designing dECM bioinks. In numerous respects, the use of porcine tissues and organs is preferable to other animals [51]. Furthermore, the use of animal tissues is more affordable and has the potential to alleviate the critical shortage of human tissue [42]. Generally, skin dECM bioink can be extracted from porcine skin and synthesized as a sponge using a decellularized process followed by the gelation process at 37 °C after printing [52]. The 3D printed skin using porcine dECM, which had a structure similar to the skin layer, shows rapid progress of re-epithelialized for chronic wound healing [52]. Nevertheless, cellular components that remained on the scaffold after decellularization may trigger an immune/inflammatory response in an immunocompetent host [53]. Overall, dECM bioinks have the capability to mimic human native tissue for clinical treatment. However, the transmission of infectious diseases and immunogenic response might be a challenge for the application of dECM bioinks [51].

### 4.2. Collagen

Many other natural-based polymers, such as collagen and gelatin, are derived from the human body. Most studies utilize gelatin and collagen as bioinks for wound healing and skin regeneration [48,49]. Collagen can be extracted from various animals’ tissues. Tissue engineering (TE) applications have employed several sources for collagen extraction, including bovine, porcine, murine, and marine [54]. However, there are religious restrictions associated with the use of bovine and porcine-derived tissue in some communities [55]. Collagen is a promising natural-based bioinks rich in natural cell-binding sites, the ability to be enzymatically degraded, and temperature-dependent gelation [56]. Few studies reported that the printability of collagen directly in 3D-bioprinting is difficult. Collagen requires a minimum of 30 min at 37 °C for crosslinking or gelation [57]. Ideally, the bioinks should be temperature sensitive and capable of faster gelation time on the printing substrate with high viscosity for printing definition [58]. Another drawback of bioprinted collagen materials is their low shape fidelity [59]. Considering the low viscosity and poor shape fidelity of collagen bioinks, the addition of cells into the bioinks resulted in an increase in the viscosity properties [56].

A 3D-printed skin implant incorporated with collagen I, fibroblasts, and keratinocytes was the first successful attempt in the year 2009 [60]. Following that study, many other experiments were using collagen bioinks and tested in animal models. The experiments demonstrated that the formulation of collagen and fibrinogen bioink printed skin implants containing cells substantially improved wound re-epithelialization as compared to control treatments [60]. By combining collagen with other polymers such as gelatin and alginate, it is feasible to develop self-supporting structures rather than entirely dependent on pure collagen [61,62]. Overall, collagen is a proven biomaterial with significant potential in regenerative medicine.

### 4.3. Gelatin

Gelatin has similar properties to collagen and is widely used for tissue engineering applications in treating damaged skin tissue. Gelatin may be derived from a variety of collagen sources. The primary commercial sources are cattle bones, hides, pig skins, and fish [63]. However, mammalian gelatin has become the most favorable source of gelatin for developing bioinks. Mammalian gelatin is produced from collagen, which is the main component of vertebrate animals’ connective tissues and bones. The exploration of two alternative gelatin sources, mostly porcine (type A) and bovine (type B) [63]. In the tissue engineering field, it is well known that gelatin has excellent potential to possess as an ideal bioinks due to its excellent biocompatibility and physicochemical properties [64]. To date, studies on the usage of single gelatin bioinks for skin tissue bioprinting are not well established. Moreover, the usage of gelatin alone in the bioprinting field is not recommended due to its temperature-dependent, reversible state from solid to gel, and it is challenging to optimize the temperature and its viscosity [49]. The temperature and viscosity of gelatin have become the main concern for the direct bioprinting approach.

In particular, pure gelatin bioinks have low viscosity if the temperature reaches 27 °C and above [64]. Therefore, gelatin bioinks work efficiently with other bioinks to achieve ideal viscosity. Gelatin with a wide range of concentrations has been utilized as a bioink with other polymers in bioprinting applications [65]. On the other hand, gelatin has low mechanical strength and no shape memory ability because it lacks persistent molecule entanglement as net points in the polymer structure [66]. Besides, chemical or enzymatic crosslinking is frequently necessary to produce excellent mechanical properties of gelatin hydrogels [67]. The thermoresponsive feature of gelatin allows it to be adjusted and physically crosslinked during bioprinting via thermal gelation, which aids in maintaining the shape of bioprinted objects [65]. A study discussed that the bioprinting of GelMA using in situ bioprinting approach successfully promotes wound closure with minimal wound contraction [68]. The study indicates that GelMA hydrogel helps to minimize wound contraction compared to non-treated wounds. 

### 4.4. Alginate

Some researchers are preferred to use alginate as bioinks for 3D bioprinting. Alginate is a natural biopolymer that is crosslinked with calcium ions to form hydrogels. It is a suitable ingredient for bioinks due to its excellent biocompatibility with human cells [69]. Apart from its remarkable biocompatibility, it is a low-cost marine material derived from brown algae’s cell walls that form a hydrogel under moderate circumstances [70]. Alginate has the great future to form gels with encapsulating cells for biological applications [71]. Moreover, alginate has high shear-thinning properties and a faster polymerization time after printing [54]. Polymerization time after printing influences the shape of the bioscaffold. The shape fidelity of the alginate hydrogel may be impaired due to its low viscosity. A low concentration of alginate frequently resulted in low viscosity of bioinks that were not stable after printing. Therefore, the pre-printing crosslinking approach was used to overcome the poor viscosity issue [69]. However, another potential disadvantage of pure alginate towards in vitro testing is that cells do not adhere naturally to the alginate hydrogels due to the absence of cell adhesion sites [72]. Therefore, it is also possible to use alginate with other polymers to improve cell adhesion and temporarily strengthen the 3D structure construct.

### 4.5. Cellulose

Cellulose (C6 H10O) is an organic molecule with a linear structure with long-chained carbohydrate polymer and glycosidic linkages [73]. The formulation of cellulose bioink is very rare. As a bioink additive, cellulose nanocrystals have been used to improve the physiological characteristics of the finished products [74]. In 3D-bioprinting, nanofibrillated cellulose (NFC) is commonly utilized as a bioink [75]. These NFCs may be extracted from either plant or bacterial ECM. Besides, carboxymethyl cellulose (CMC) is derived from cellulose, a naturally occurring, biocompatible, biodegradable, and abundant biopolymer [76]. Because of its high solubility in water and numerous carboxyl groups, CMC is an excellent option for the preparation of hydrogels. The printability of NFC and CMC bioinks as an agent of wound dressing becomes more excellent after being incorporated with alginate [77]. The NFC-CMC-alginate possesses a favorable environment towards many cells.

### 4.6. Silk

In another approach, silk has been utilized as a promising biomaterial for wound healing. Silk generated by silkworms and spiders has been widely researched for a variety of biological uses [78]. Silkworm silk is composed of a core of two proteins from the fibroin family and a covering of proteins from the sericin family [79]. Apart from the silkworm, spiders utilize silk threads to construct webs, capture prey, and move [78]. Silks have subsequently been investigated for applications in tissue engineering. Both silkworm and spider silks have proven to assist the adhesion and proliferation of a wide range of cell types [80]. Based on an aqueous solution system, the silk fibroin (SF) can be further processed into different types of materials in films, particles, fibers, sponges, and hydrogels. However, there is a barrier hindering 3D printing fabrication in SF bioink that is caused by low concentrations and viscosity [74,75]. Increasing its concentration and adding other high viscosity additives are perhaps useful strategies in improving its printing processability and biofunction ability [81]. However, the viscosity of the SF bioink depends on the type of 3D-bioprinter used. In the case of an extrusion-based printer, a high viscous solution is needed for the extrusion, whereas in the case of an inkjet printer, the viscosity must be low in order to generate droplets [82].

Besides, considering the function of SF biomaterial in tissue construction, bioink should be designed extensively. The addition of other biomaterials with SF could enhance the properties of the bioinks. A study on the incorporation of gelatin with SF for in vivo wound healing treatment demonstrated that the fibroblasts migrated from the wound bed, and granulation was initiated as the defect site was replaced with regenerating tissues [83]. On the other hand, the modulation of the physical properties of SF is time-consuming and incompatible with bioprinting. To resolve these issues, a composite hydrogel made of SF and sodium alginate (SA) was studied for possible application in bioprinting [84]. The result demonstrated that bioink made from SF-alginate is suitable for 3D bioprinting due to its fast gelation, cell-loading capacity, and lack of cytotoxicity. Thus, the biocompatibility of the SF bioink can be considered for future biomedical applications.

### 4.7. Fibrinogen

Fibrin has been extensively employed in the tissue engineering field due to its biocompatibility, biodegradability, and adjustable mechanical and nanofibrous structural characteristics [85]. Fibrinogen is an abundant plasma protein that is fluidic while circulating in the bloodstream but turns into a natural hydrogel-like matrix by the action of thrombin [86]. Fibrinogen is a precursor of fibrin [87]. Fibrinogen is a viable bioink component for skin bioprinting as it is a natural part of the wound healing process. The source of the fibrinogen can be obtained from bovine plasma (65–85% protein, 75% clottable protein) with MW of 63.5 kDa and 56 kDa for α-chain and β-chain, respectively [88]. The current trend of protein-derived bioinks provides an excellent potential for wound healing treatment. Along with the low viscosity properties, the fibrinogen bioink is more suitable to be used for mixing cells via the conventional 3D scaffolding approach [85]. To address this issue, hyaluronic acid, fibrinogen, and collagen type I were all formulated as bioink utilized for the in vitro studies [47]. Generally, the gelation of the fibrinogen will be induced by the presence of thrombin. The in vitro study demonstrated that DFs embedded in the fibrin-based sheets had >90% viability based on a live/dead assay performed after 10 days in culture.

**Table 1:** Properties of natural-based bioinks.

Type of Bioinks	Sources	Properties	References
DECM	Majority composed of ECM	dECM-based bioinks have viscoelastic behavior and rheological properties of dECMs, including shear viscosity and shear modulus that can preserve cells during printing. Besides, it is a biodegradable and low cytotoxicity biomaterials.	[89,90]
Collagen	Bovine, porcine, murine, and marine	Low viscosity, high shear stress, low viscosity, and weak mechanical strength.	[58,63,91]
Gelatin	Bovine, porcine	Has controllable mechanical properties depending on the concentrations, temperature-dependent, reversible state from solid to gel, and its challenging to optimize the temperature and its viscosity	[52,92]
Alginate	Algae	has high shear-thinning properties and a faster polymerization time after printing. However, alginate do not have cell adhesion sites	[57,72,74]
Cellulose	Plant or bacterial ECM	Naturally occurring, biocompatible, biodegradable, and abundant biopolymer, high solubility in water and numerous carboxyl groups	[78,79]
Silk	Silkworms and spiders	low concentration and viscosity, slow biodegradation rate	[74,75,80]
Fibrinogen	Plasma protein	Biocompatibility, biodegradability, adjustable mechanical properties, nanofibrous structural characteristics, and low viscosity properties	[87,88]
Chitosan	Chitin	Biocompatibility, antibacterial properties, thermosensitive, and low mechanical strength	[93,94,95]

### 4.8. Chitosan

Chitosan and dibutyrylochitin are natural polysaccharides that are derived from chitin and are highly used as a new generation of biomaterials [96]. The presence of chitosan in the scaffold enables strong antibacterial activity, water, and protein absorption capability and maintains the moisture and nutrition balance to avoid wound infection [93]. Generally, as the application of chitosan as a biomaterial has been widely exploited, their low mechanical strength property may limit their application for tissue engineering [97]. To address this limitation, this polymer needs to be crosslinked or combined with other natural/synthetic polymers to enhance mechanical strength [94]. Chitosan scaffold crosslinked with genipin has been reported to have good biocompatibility with excellent mechanical strength [95]. Genipin has extensively replaced glutaraldehyde and other crosslinkers but has the advantages of stability, biocompatibility, well-defined chemistry, and general safety of the products [98]. Besides, the photocrosslinking approach has emerged as an alternative for crosslinking method for chitosan [99]. In a 3D-bioprinting application, the printability of chitosan has been evaluated at low temperatures for printing due to its thermosensitive hydrogel properties [94]. 

Generally, research has demonstrated that chitosan help to speed up skin wound healing by stimulating the formation of inflammatory cells (represented by macrophages), fibroblasts, and capillaries. Chitosan can stimulate the release of cytokines such as transforming growth factor- (TGF-), PDGF, and IL-1 in macrophages [100]. In vitro cell viability studies have been evaluated to observe the live/dead cell trend of dermal fibroblasts against chitosan, proving 88.5% of cell viability after performing bioprinting [101].

## 5. In Vivo and In Vitro Studies

Bioinks should promote cell proliferation and produce a substrate with mechanical and physical characteristics similar to the native environment [82]. Human skin tissue engineering has been introduced to mimic normal structural and functional aspects of the natural human skin. Therefore, in vitro and in vivo studies are crucial to evaluate the effectiveness of the custom bioinks towards human skin cells to promote wound closure for human application. In this regard, in vitro studies enable not only the examination of fundamental skin processes but also the risk evaluation of diverse chemical substances that are topically administered to the skin without the use of animal models [102]. Usually, results obtained from animal models are frequently significantly limited due to different variations in metabolism and anatomical architecture compared to human skin.

### 5.1. Cell Viability and Proliferation

The cell proliferation rate is a reliable assessment for in vitro study to evaluate the survivability of the biological cell after printing. The encapsulated cell in hydrogel has many growth limitations, including restricted cell connections due to the entrapment of the cells within the hydrogel [103]. Cell viability and proliferation rates have been identified as good selective indicators for cell sustainability. In vitro cellular assessment offered inexpensive and rapid approaches without using animal models for testing indicators [104]. Currently, there is a broad spectrum of cell viability assays available in the market, including qualitative and quantitative evaluation. A study of alginate-gelatin bioinks has been conducted to evaluate the cellular behavior for in vitro and in vivo applications. The DFs showed more than 95% cell viability after day three and successfully increased the proliferate rate until day 14 [105]. Besides, an MTT study was employed to investigate the viability of DFs and KCs towards chitosan-genipin-Polyethylene glycol (CH-GE-PEG) hydrogel as a cell carrier after printing [101]. The study reported that the CH-GE-PEG bioinks are biocompatible with DFs and KCs with more than 88% cell viability. According to Hafezi and co-workers (2020), the porosity and permeability of the hydrogels may influence the cell viability and proliferation rates [101]. The porous structure of hydrogel will allow the permeability for nutrient transportation and removal of waste products that could give a toxic environment to the cells.

Besides, dECM-collagen bioinks demonstrated excellent biocompatibility results for DFs and KCs with more than 90% cell viability [42]. The proliferation rate for both DFs and KCs was well-established after day 14 during in vitro study. However, Baltazar and co-workers (2020) conducted a preliminary experiment on collagen bioinks only for 3D-bioprinting encapsulated cells of DFs and KCs [106]. The bioprinted constructs indicate excellent improvement distribution of DFs and KCs and improved morphology of the printed cells. A study involving biomimetic bioinks has been performed with a combination of fibrinogen-dECM towards fibroblasts cells. This combination successfully shows excellent cellular activity compared to fibrinogen bioinks only [50]. The dECM helps to provide a natural microenvironment to the cells, while the fibrinogen will enhance the secretion of growth factors to promote cell growth [86]. 

Apart from the fibrinogen, bioprinting with fibrin-based bioinks also indicates a new trick for enhancing the cellular activity in skin tissue bioprinting. The fibrin is derived from fibrinogen and is suitable to be combined with cells and other bioactive compounds for rapid wound healing treatment [107]. The printing of DFs and KCs using fibrin bioinks provides an optimum condition for cellular growth of DFs compared to KCs [104]. This result occurred due to the improper formulation of the fibrin bioinks for KCs. The stiffer surface of the fibrin hydrogel will slow down the proliferation rate of the KCs [108]. The slow proliferation of cells is highly correlated with the stiffness, porosity, and topography of the hydrogels. Stiffer hydrogels have more rigid structure. A sufficient stiffness of hydrogel is required as an effective treatment for skin wound healing. However, it has not yet been investigated how the stiffness of the matrix may impact wound healing in vivo [109].

Besides, studies on gelatin-based bioinks only for encapsulated skin cells by using 3D- bioprinting have not been discovered yet. However, a study on the cellular proliferation rate for DFs and KCs against silk fibroin-gelatin bioinks gradually proven that the DFs and KCs indicate excellent cellular growth from day seven until day 21 [103]. The combination of silk fibroin-gelatin promotes cell adhesion site compared to silk fibroin only. This study can be supported by the finding of the 3D printed gelatin-based silk fibroin (SF) scaffolds aided in the repair of a full-thickness skin lesion in vivo model. The formulation of the SF-gelatin bioinks with FGF-2 might improve treatment efficacy even further [83]. The immobilized growth factor FGF-2 was shown to have sustained release kinetics and to induce cell proliferation and migration in vitro. The in vivo model demonstrated that the epithelisation process occurred in the treatment groups compared to non-treatment groups. Therefore, the silk fibroin derivative bioinks have shown tremendous promise for treating such severe skin defects.

### 5.2. Cell Migration

**Figure 8:**
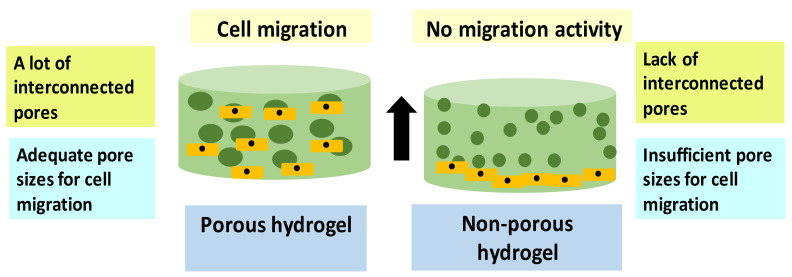
A comparison between cell migration activity in the porous and non-porous hydrogel.

Many studies have evaluated the association of cell migration activity against scaffold pore sizes after 3D-bioprinting technique. The cell migration test is required for specific biological cell activities such as tissue maturity progress and metastasis activity [110]. Several research findings suggested that the pore size of the scaffold could influence the cellular activity behavior, including cell migration and cell proliferation rate [111]. The pores distribution of the scaffold allows the cells to penetrate and be well-differentiated throughout the pores [112]. Figure 8 shows the illustration of cell migration activity in the porous and non-porous hydrogel. Hydrogels are the scaffold fully hydrated network with many advancements in the fabrication field and applications. The advancement in 3D-bioprinting technology provides an opportunity to develop a uniform and porosity scaffold required for tissue regeneration [113].

Nowadays, there are several approaches exist for the determination of scaffold pore sizes. However, with the development of computer software and imaging technology, the most widely used technology for porosity evaluation is by using a scanning electron microscope (SEM) and micro-computed tomography (micro-CT) [114]. The scaffold porosity will be determined based on several pore characteristics, including open pores sizes, shape, pore distribution, and mutual interconnection [114]. The most appropriate scaffold pore sizes that can support cellular activities and nutrient transportation are between 100 to 300 µm [115]. The SEM pore sizes analysis tends to have a greater depth of field, high resolution, and high magnification range than other computed technologies [116].

Generally, different formulations of bioinks have demonstrated different cellular activity, as summarized in Table 2. In wound healing, new tissue development will begin within 2 to 10 days after the lesion and consists of cell proliferation and the migration of diverse cell types [11]. Collagen bioinks have been reported to support cellular migration activity by synthesizing a number of ECM proteins for new tissue formation [46]. This can be supported by a previous study on encapsulated DFs, KCs, and melanocytes in collagen bioinks. In a study conducted by Hu and co-workers (2021), it was seen that the collagen hydrogel has adequate pore sizes for cell distribution activity [117]. Collagen-based bioinks suffer from poor mechanical strength and possess a drastic shrinkage after two to three weeks in vitro testing [118]. 

Moreover, Admane and co-workers (2019) have developed new fabrication strategies by using silk-gelatin bioinks [118]. The silk-gelatin bioinks were filled in with the DFs and KCs prior to bioprinting. The researchers successfully identified both DFs and KCs were distributed evenly in the printed bioscaffold. The keratinocytes were also seen to migrate throughout the pores after day 7 until day 14. Fibrinogen-based bioinks have been tested in vivo for wound healing treatment on mice by using skin cells [86]. This work demonstrated the feasibility of using fibrinogen-based bioinks to generate skin tissue that was effectively remodeled in vivo. Besides, the new formulation of bioinks by combining fibrinogen with dECM could enhance the migration activity of fibroblasts cells. This is due to the porous structure of fibrinogen-dECM hydrogel that allows cell movement throughout the pores [50].

### 5.3. Protein/Gene Expression Evaluation

In the biopharmaceutical field, the evaluation of protein expression for mammalian cells has become an important parameter of its products, including reagents, vaccines, and drugs for therapeutic and in vivo uses [119]. Protein expression refers to how protein is synthesized, modified, and undergoes a regulatory process in living organisms. The proteins produced by the mammalian cell lines, such as monoclonal antibodies, will develop protein folding and post-translational modifications that contribute to biological activity [120]. Immunocytochemistry (ICC) and immunohistochemistry (IHC) are visualization techniques that require antibodies to recognize an antigen that presents in the tissue [121]. The evaluation of the IHC results is determined according to the different color intensities of stained cells.

The finding indicates there are significant differences between different concentrations of hydrogels against the inflammatory expression. Hydrogels with high concentrations of HA-m-tyrosine and collagen have good biocompatibility and low inflammatory responses as the CD4 and CD8 did not stain. In contrast, gene expression is a complex process that involves many synthesis levels. The production of gene phenotypes is mainly derived from the protein synthesis process. Several processes are needed for complete gene expression, such as transcription, translation, and turnover of mRNAs and proteins [122]. Reduction in gene expression might contribute to significant implications resulting in multiple types of diseases [123]. Gene expression quantification helps to observe any reactions and changes in the cells’ gene expressions towards the bioscaffold geometry [124]. 

In 3D-bioprinting, hydrogel was used as a medium to deliver cells and enhance tissue regeneration. However, the materials of the scaffold can also interfere with cellular activities. Wound healing is an evolutionarily conserved, complicated, and multicellular process needed to restore the structural and functional integrity of the skin. A dynamic signaling network including multiple growth factors, cytokines, and chemokines executes and regulates the intricate process [46]. A comparative study was evaluated on gene and protein expression quantification of the fibrinogen-based bioinks using KCs [118]. Initially, the KCs demonstrated an 800-fold rise in fibronectin gene expression on day 7, a 900-fold increase on day 14, and a 1600-fold (*p* < 0.001) increase on day 21 compared to day 7 and day 14. The fibronectin gene expression is significantly related to the scarless wound healing end product. Therefore, less scar formation will occur after tissue remodeling. Moreover, fibronectin is a kind of protein that can stimulate the production of IL-4 to accelerate wound closure [125]. Apart from that, the dECM is known as a bioactive material for wound healing treatment due to its ability to provide a favorable microenvironment for skin cells. Furthermore, gene and protein expression of normal skin fibroblasts on dECM-based scaffolds revealed that the dECM-based scaffolds have the potential to enhance the production of extracellular matrix proteins [89]. 

Interestingly, dECM bioinks provide a great contribution to reducing scar formation. A study was conducted to evaluate the integrity, tissue healing, and epithelialization to see how dECM affected gene expression of keratinocyte growth factor-1 (KGF-1) [42]. The result indicates that the skin progression is associated with the production of skin ECM (collagen type I, fibronectin, decorin, collagen type III). The HDFs-laden 3D constructions were grown for 14 days using dECM bioink, and the gene levels were compared to those of the collagen group. The results showed that the levels of all genes were greater in the 3D cell-printed structures utilizing dECM bioink than in the collagen group. It indicates that the dECM bioink has higher cell potency, which likely leads to improved tissue development in vitro and in vivo. This finding might be attributed to the preservation of the skin-specific ECM microenvironment in the dECM bioink. Thus, Table 2 summarized the effect of cellular activity study of skin cells towards different type of bioinks.

**Table 2:** Effect of cellular activity study of skin cells towards different type of bioinks.

Type of Bioinks Used for Tissue/Organ Construction	Human Skin Cells	Study Methods	Cell Proliferation/Viability	Cell Migration	References
dECM and commercial collagen type-I	DFs and KCs	In vitro and in vivo	>90% cell viability. DFs and KCs demonstrated well proliferation rate after 14 days.	Not-reported	[42]
Fibrin	DFs and KCs	In vitro	The number of cell viability for DFs much higher than KCs.	Enhance cell migration	[104]
Silk fibroin-gelatin	DFs and KCs	In vitro	Excellent cell growth	Intensive migration of cell on day 7 onwards.	[103]
Collagen	DFs and KCs	In vivo	Well-distributed of DFs and KCs after day 30 observation.	Not-Reported	[106]
Chitosan-Genipin-Polyethylene Glycol (CH-GE-PEG)	DFs and KCs	In vitro	>88% cell viability	Not-Reported	[101]
Bacterial cellulose (BC)/acrylic acid (AA)	DFs and KCs	In vitro	The cell viability for the hydrogel significantly increased from day 1 to day 7.	No cell migration activity has been detected from day 0 to day 7 as the cell is only static on top of the hydrogels.	[126]
Fibrinogen-dECM	Fibroblasts	In vivo	Fibrinogen-dECM hydrogel indicated improvement in cellular activity compared to fibrinogen hydrogel only.	Fibrinogen-dECM hydrogel has a porous structure hydrogel which allows cells migration and movement throughout the pore	[50]
GelMA-PEO	Fibroblasts	In vitro	The combination of GelMA-PEO enhances the cell viability and proliferation rate compared to pure GelMA.	A lower concentration of GelMA bioinks can enhance cell migration through the pores	[64]
Alginate-gelatin	Fibroblasts	In vitro	>95% cell viability after day 3 followed by increasing in the proliferation rate until day 14	Not-Reported	[105]
Chitosan/carbon dots (CDs)	Fibroblasts	In vivo	Cell viability evaluation shows no significant difference between the fibroblasts and the chitosan. This indicates that chitosan/CDs have high biocompatibility and are non-toxic to the cells.	Not-Reported	[127]
Alginate/Gelatin/Silver nanoparticle	Fibroblasts	In vitro and in vivo	Reduction in the wound area in a mouse model from day 3, day 7, and day 14.	Not-Reported	[128]
Biliverdin/Silk Fibroin (BVSF)	Fibroblasts	In vitro and in vivo	BVSF hydrogel treated wound showed the smallest area among all groups on day 3, 6, 9, and 13 in a mouse model. Besides, BVSF hydrogel had an approximate 50% wound closure, which is about 20% better than the SF group and 40% better than the control group on day 6.	BVSF stimulated cell migration	[129]
Alginate with hydrogen sulfide (H_2_S)	Fibroblasts	In vivo	Alg/H_2_S, 0.5% hydrogel, resulted in the highest wound closure in a rat model with 98 ± 1.22%, which was statistically significant in comparison with the negative control group. Hence, Alg/H2S 0.5% hydrogel provided the best treatment, which absolutely required to be confirmed with microscopic and histopathological observations.	Favorable for cell migration activity	[130]
Collagen	DFs, KCs, and melanocytes	In vitro	50,000 KCs lead to the formation of a thin epidermal region. 75,000 KCs lead to the formation of a medium epidermal region. 125,000 KCs developed a thick epidermal region.	Adequate pore sizes for cell distribution.	[117]

To date, the use of collagen bioinks improve the cellular activity of the skin cells to promote skin regeneration. An evaluation of the skin cells’ expression after seeding on the collagen hydrogel shows the keratinocytes’ migration activity in the injured skin [131]. The migration activity of the cell was influenced by the presence of integrin and matrix metalloproteinases expression. Besides, a study on the polyacrylamide gel coated with collagen I indicate the KCs respond to IFN Ƴ responsive genes (HLA DR and CXCL10) [132]. The presence of these genes promoted the excellent expansion of KCs for wound healing. Besides, the findings of a previous study showed that collagen also modulates the wound microenvironment and controls the inflammatory response preparing for wound closure [133]. Moreover, the study demonstrated that numerous mature collagen fibers were seen with higher in collagen type I compared to collagen type III to promote tissue formation. 

Furthermore, using silk fibroin and collagen hydrogel revealed the regeneration of the epidermis skin layer as wound healing treatment in mice model after 21 days of treatment [134]. This is due to the presence of suprabasal keratin marker cytokeratin 10 (CK10), basal keratin marker cytokeratin 14 (CK14), and involucrin (INV) for keratinocyte differentiation in both types of hydrogels. Besides, an in vitro study was evaluated to compare the effectiveness of silk and collagen for wound healing treatment. The study reveals that KCs grown on a silk fibroin surface had greater relative gene expression of epithelialization markers KRT1, TGM3, and IVL than HEKs cultured on TCPS or a collagen surface [135]. This tendency indicated that HEK develops from spinous cells to cells on the top layers of the silk fibroin surface and the stratum granulosum. The relative gene expression shows that HEK can more efficiently accelerate skin regeneration on silk materials than on collagen materials.

Apart from that, a combination of collagen-hyaluronic acid showed that the hydrogel released the heparin binding-epidermal growth factor (HB-EGF) used as wound dressing [136]. This finding can be supported by the in vivo study where the inclusion of HB-EGF resulted in an increase in granulation tissue compared to wounds treated with fibrin alone after one week of evaluation [137]. Thus, the presence of HB-EGF may accelerate skin re-epithelization. Overall, based on these interactions of bioinks with skin cells, the majority of bioinks assist skin cells in releasing specific indicators that can improve wound healing therapy.

## 6. Conclusions and Future Perspectives

In conclusion, 3D-bioprinting has been accepted as a novel treatment to solve chronic wound healing issues. Several types of bioprinting techniques are available, including extrusion-based, laser-assisted, and inkjet bioprinting techniques. However, different biomaterials have different printing requirements that need to be conducted with specific bioprinting techniques. The main components for 3D bioprinting are the bioinks composed of biomaterials, cell selections, and crosslinkers to enhance mechanical strength. Skin tissue is a complex structure and consists of different types of cells. Tissue vascularization is essential for oxygen and nutrient transportation for a living cell. The usage of bioinks and 3D-bioprinting provides more opportunities to develop scaffold and in vitro models that highly mimic human native ECM. The selection of the bioinks is very crucial in influencing cellular activity. The bioinks must be able to provide a micro-conducive environment for cells. For in vitro models, the most common cellular activities being evaluated are cell viability, rapid proliferation rate, and migration activity. Excellent bioinks must possess a high percentage of cell viability, proliferation rate, and a highly porous structure model that allows cell migration activity throughout the pores. Besides that, the protein and gene expressions quantification for the cells are crucial to determine the syncretization and modification of the cell. In this review, the cellular interactions of the human skin cells, DFs and KCs, have been discussed according to different types of natural-based bioinks. Based on the findings, a combination of dECM with collagen type I are the most favorable bioinks as they provides a conducive environment for both DFs and KCs cellular activities. However, other natural polymers such as silk-fibroin and collagen provide excellent cell distribution due to their porous structure 3D model. 

Although 3D bioprinting technology has become an important topic for several decades, the investigation of the printing technique for skin tissue is still recent and new. Nevertheless, the flexibility of 3D-bioprinting allows a versatile system for 2D and 3D tissues engineering applications. A substantial amount of research has been gathered numerous advantages that 3D models systems offer over standard 2D cell cultures. With advancements in 3D bioprinter precision, accuracy, and scale, they may become ideally suited for usage throughout skin graft development. This review discussed the designation of in vitro model and the cellular activity study of skin cells as a treatment for chronic wound healing. However, the in vivo assessment for 3D bioprinting is still at the initial stage of the investigation with a lack of output. There are numerous challenges that need to be overcome before they can be used in therapeutic settings, notably as an in situ direct application. Therefore, in the future, we recommend using both natural-based and synthetic polymers for in vivo study of different tissues/organs development via a 3D bioprinting approach.

## Data Availability

The data presented in this study are available on request from the corresponding author.
